# Changes in distress and turnover intentions among hospital‐based nurses working during the first 8 months of the COVID‐19 pandemic in Denmark: A prospective questionnaire study

**DOI:** 10.1111/jonm.13781

**Published:** 2022-09-27

**Authors:** Berit Kjærside Nielsen, Caroline Trillingsgaard Mejdahl, Morten Deleuran Terkildsen, Mimi Mehlsen

**Affiliations:** ^1^ Department of Public Health and Health Services Research DEFACTUM Aarhus Denmark; ^2^ Department of Forensic Psychiatry Aarhus University Hospital Psychiatry Aarhus Denmark; ^3^ Department of Psychology and Behavioural Sciences Aarhus University Aarhus Denmark

**Keywords:** COVID‐19 pandemic, follow‐up survey, nurses, psychological distress, turnover intentions

## Abstract

**Aim:**

To describe changes in distress among Danish hospital‐based nurses during the early month of the COVID‐19 pandemic and to examine predictors of distress and turnover intentions.

**Background:**

Outbreak of infectious diseases such as the COVID‐19 pandemic can increase the likelihood that health professionals suffer from poor mental health even after the outbreak.

**Methods:**

A prospective study among 426 Danish hospital‐based nurses during the early month of the pandemic. Participants completed self‐administered questionnaires regarding mental health and COVID‐19 worries, as well as turnover intentions.

**Results:**

Nurses with brief work experience reported higher increase in distress. Feeling unsafe at work, having low trust in management and being anxious for relatives were associated with increased distress. Finally, feeling unsafe at work, being anxious for relatives and having low trust in management were predictors of intention to change job.

**Conclusion:**

This study suggests that the subjective experiences of uncertainty in work during the COVID‐19 pandemic have more impact on nurses' distress than COVID‐19 related conditions at hospitals. Finally, the study provides empirical support for the association between COVID‐19‐related worries and turnover intentions.

**Implication for nursing management:**

Knowledge of risk factors for psychological distress as well as predictors of turnover intention is necessary and may provide nurses and health‐care systems with the ability to respond better against future pandemics and to retain nurses in the organization and in the profession.

## 1 INTRODUCTION

In December 2019, in Wuhan, China, an unknown and potentially life‐threatening disease caused by SARS‐CoV‐2 was discovered. The disease was named Coronavirus disease 2019 (COVID‐19) and is a viral respiratory infection. COVID‐19 spread rapidly throughout the world, and on March 11, 2020, the World Health Organization declared COVID‐19 a pandemic (WHO, 2020). Health professionals are under physical and psychological pressure when facing largescale health crises such as COVID‐19. Studies from previous pandemics have found that these events can lead to extraordinary pressure on health professionals (Liu et al., 2012). One of the major problems health professionals faced during the onset of COVID‐19 was the uncertainty associated with the disease arising from the different ways of transmission of the virus, uncertainty regarding protective measures and the difficulty in implementing physical distancing in clinical practice. Moreover, factors such as increased workload, lack of protective equipment, physical exhaustion, risk of infection and ethical issues can significantly affect health professionals' physical and mental state (Chen et al., 2020; Vizheh et al., 2020). Poor mental health can manifest in adverse psychological reactions such as depression, anxiety, stress or insomnia, collectively termed distress. Negative aspects of work‐related distress include burnout and feelings of personal and professional inadequacy (Maslach et al., 2001). It has been demonstrated that health professionals are particularly vulnerable to developing work‐related distress during pandemics (Lung et al., 2009; Wu et al., 2009). Moreover, studies have found that nurses represent the group among various health professions where the prevalence of adverse psychological reactions is highest (Benfante et al., 2020; Luo et al., 2020; Pappa et al., 2020; Sanghera et al., 2020; Vizheh et al., 2020). One of the first studies investigating mental health among health professionals in Wuhan during COVID‐19 identified adverse psychological reactions such as anxiety, stress, depression and sleep problems (Lai et al., 2020). The same pattern has since been found in several studies (Cabarkapa et al., 2020; Krishnamoorthy et al., 2020; Luo et al., 2020; Pappa et al., 2020; Salari et al., 2020; Sanghera et al., 2020; Vizheh et al., 2020). Since the emergence of the COVID‐19 pandemic, nurses all over the world have had major work tasks, and while the majority have been dedicated to the job, the extraordinary pressure brought on by the pandemic on health‐care systems worldwide may affect well‐being and work performance. A study among nurses in Danish hospitals during the first wave of the COVID‐19 pandemic showed that mental health was strained (Mejdahl, Mehlsen, et al., 2022; Mejdahl, Nielsen, et al., 2022). In order to be able to carry out one's work tasks in an appropriate manner during major health crises such as COVID‐19, it is necessary for nurses to uphold good mental health (Catton, 2020; Mo et al., 2020). However, COVID‐19 related distress may lead to long‐term effects on nurses' job satisfaction and, ultimately, turnover (Labrague & de Los Santos, 2021). Turnover intention is argued to start with psychological reactions to negative aspects of organizations or jobs, but the relationship and the mediating effects among variables related to turnover are complex (Hayes et al., 2012). Negative psychological reactions related to COVID‐19 could therefore lead to long‐term effects on nurses' work performance and satisfaction, which may initiate thoughts of leaving one's job (Labrague & de Los Santos, 2021). The International Council of Nurses has underlined the unfortunate impacts of the COVID‐19 situation on the nursing workforce, including the potential to increase burnout and increase the number of nurses leaving the profession (James Buchan, 2020). In Denmark, a 36% decrease in applicants for nursing education is recently documented (Dahlmann, 2022).

Potential long‐term distress and intention to turnover experienced by nurses is vital to investigate, but most of the existing studies on nurses' mental health during COVID‐19 are cross‐sectional and have been conducted in contexts other than Danish. Insight in local nurses' reactions to the pandemic is critical to inform and develop the local support of nurses during and after the pandemic. Therefore, the present study aimed to analyse changes in symptoms of depression, anxiety, stress and sleep problems among hospital‐based nurses during the first 8 months of the pandemic in Denmark and to examine what predicted (a) increased distress and (b) turnover intentions.

## 2 METHODS

### 2.1 Study design

A prospective questionnaire was conducted among Danish hospital‐based nurses during the first 8 months of the COVID‐19 pandemic. In addition, a small sample also participated in in‐depth interviews. Results from the qualitative study have been published elsewhere (Mejdahl, Nielsen, et al., 2022).

### 2.2 Study sample and data collection

Participants were recruited through targeted advertising on social media. We invited hospital‐based nurses in Denmark to participate in a survey investigating nurses' mental health during COVID‐19. The survey was to be answered electronically via SurveyXact and was available from 18 May 2020 until 7 June 2020. The only criteria for participating was currently working as a hospital‐based nurse with sufficient mastery of Danish to complete a questionnaire. A follow‐up survey was sent to those who agreed to receive a second questionnaire, which was available from 30 November 2020 until 31 December 2020. Technical procedures regarding answering the online questionnaire and the understanding of the individual questions were tested among a small group of nurses prior to the launch.

### 2.3 Measures

The questionnaire consisted of demographic questions (sex, age, region of employment, years working as a nurse and ward type), four internationally validated scales measuring mental health and seven single‐item questions related to the participant's specific experience with and concerns regarding COVID‐19.

The primary outcome measures were the occurrence of depressive symptoms, anxiety, insomnia and stress. *The Patient Health Questionnaire* (PHQ‐9) was used to determine the presence of depressive symptoms (Kroenke et al., 2001). PHQ‐9 consists of nine statements, where the respondent indicates on a 4‐point Likert scale the extent to which they have been bothered by a problem within the last 2 weeks. Then a total score of between 0 and 27 is calculated, with a higher score indicating more depressive symptoms. PHQ‐9 has proven to be a valid and reliable questionnaire but has not been validated in Danish (Martin et al., 2006). Cronbach's alpha in the present study was .86.

*Generalized Anxiety Disorder* (GAD‐7) was used to measure anxiety symptoms (Spitzer et al., 2006). GAD‐7 consists of seven questions in which the respondent on a 4‐point Likert scale evaluates the frequency of anxiety symptoms within the past 2 weeks. A total score is calculated with values from 0 to 21, where a higher score indicates a higher degree of anxiety. Thus far, there are no validation studies of the Danish version of the GAD‐7 scale, but it is widely used in Danish populations. Cronbach's alpha in the present study was .89.

*The Insomnia Severity Index* (ISI‐7) includes seven questions that measure the severity of insomnia seen over the past 2 weeks (Bastien et al., 2001). The respondent evaluates on a 5‐point Likert scale the degree of difficulty in falling asleep, staying asleep, waking up early, satisfaction with current sleep and the degree of worry or anxiety caused by difficulty sleeping. The overall score goes from 0 to 28, a higher score indicating greater insomnia severity. Preliminary validation of the Danish translation of ISI‐7 has been conducted, showing good psychometric properties (Dieperink et al., 2020). Cronbach's alpha in the present study was .92.

Finally, we included the *Perceived Stress Scale* (PSS‐10), a general measure of perceived stress that expresses how a person experiences their life as unpredictable, uncontrollable and stressful (Cohen et al., 1983). The PSS‐10 consists of 10 items, and the respondent is asked to rate feelings and thoughts within the last month. PSS‐10 has been translated and validated in a Danish context and used in several Danish studies, including the National Health Profile (Eskildsen et al., 2015). The PSS‐10 score ranges from 0 to 40, a higher score indicating a higher degree of perceived stress. Cronbach's alpha in the present study was .89.

#### 2.3.1 COVID‐19 items

The COVID‐19 single‐item was developed based partly on items used in one of the first studies involving frontline health professionals (Lai et al., 2020) and partly on a dialogue involving hospital‐based nurses, who, 1 month into the pandemic in Denmark, had practical experience of working after the outbreak. The COVID‐19 items consisted of seven questions concerning work‐related worries regarding COVID‐19. Four questions were asked to the participants in both surveys, whereas three questions concerning flexibility, recognition and consideration of changing jobs were only asked in the second survey (see Table 2). Results based on cross‐sectional data from the first survey have been published elsewhere (Mejdahl, Mehlsen, et al., 2022). In the following, consideration of changing jobs is labelled turnover intentions, defined as an employee's intention to change jobs voluntarily. If the participants answered yes to the question, they were asked to elaborate on the reasons in an open text box.

### 2.4 Data analysis

The age of the participating nurses was calculated based on their reported date of birth. Seniority was based on the number of years the respondent reported working as a nurse. On the scales, missing items resulted in incomplete scores for 12–19 participants. Missing data were treated with listwise deletion. To examine if changes in distress differed between subgroups of nurses, we conducted a series of mixed analyses of variance (ANOVAs), testing the time x group interaction. These subgroups included nurses reporting long and short work experience, being high and low on their ratings of being anxious for relatives, feeling unsafe at work, having trust in management, caring for COVID‐19 patients and being deployed to another ward. Emerging evidence suggests that less experienced nurses have an increased risk of stress, depression and burnout during COVID‐19 (Sriharan et al., 2021). Nurse seniority (0–2 years nurse vs. > 2 years nurse) was therefore viewed as a subgroup. The COVID‐19 questions relating to being anxious for relatives, feeling unsafe at work and having trust in management were recurring themes in the qualitative statements in the open text box and provided in‐depth perspectives of why and how the work as a nurse gave rise to distress (data not shown). Therefore, the themes were considered indicators of COVID‐19‐related job burden together with having cared for COVID‐19 patients and being deployed to another ward. We grouped the nurses based on their responses to the COVID‐19 having feeling unsafe at work, being anxious for close relatives and having trust in management. For the questions concerning feeling unsafe and feeling anxious for relatives, we created a highly unsafe and highly anxious category to compare the most extreme responders with the other groups. For the trust in management question, this was not feasible because only 3% reported the most extreme answer ‘No trust in management’. Therefore, we chose to collapse this group with the group answering ‘Little trust in management’.

Finally, to test the combined effect of these factors on distress in a multivariate analysis, we conducted a series of linear regressions including the above variables as independent variables with follow‐up stress, depression, anxiety and insomnia as dependent variables, while controlling for the baseline level of the respective distress measure. We also tested combined predictors of turnover intentions in a binary logistic regression. The significance level was set at *p* < .05. Data were processed and analysed in SPSS version 27.

### 2.5 Ethical statements

According to Danish law, this kind of self‐reported questionnaire study does not require notification to the Committee on Biomedical Research Ethics. The study followed the ethical principles of research involving people, as set out in the Declaration of Helsinki. Data were collected anonymously via a web‐based, public questionnaire link. Participation was voluntary. Written information about the study, including contact information for the research leader, accompanied the study. The study was registered in the Central Denmark Region's research notification system (journal number: 1‐16‐02‐193‐20). The participants gave electronic informed consent to participate and did not receive any financial compensation.

## 3 RESULTS

A total of 1165 completed the baseline survey (Mejdahl, Mehlsen, et al., 2022) and of those, 426 (36.6%) completed the follow‐up 6 months later and were included in the present study (see Table 1). Compared to those only participating in the baseline survey (*n* = 739), the participants in the follow‐up were more likely to be older (*p* < .05). Concerning years working as a nurse or levels of distress measured at baseline, no statistically significant differences were found. The number of nurses deployed to other departments was the same in the baseline‐only and the follow‐up sample, whereas nurses working in outpatient clinics or intensive care units were more likely to participate in the follow‐up survey than those who only participated at baseline (*p* < .05). Feeling unsafe because of COVID‐19 at baseline was the same in the baseline‐only and follow‐up sample (*p* > .05), but the participants in the follow‐up reported at baseline were more concern about their relatives and had less trust in the management's handling of COVID‐19 (*p* < .001).

**TABLE 1:** Participant characteristics

Variables	Baseline sample (*N* = 1165)	Follow‐up sample *N* = 426 (100%)
**Sex**		
Male	38 (3%)	16 (4%)
Female	1125 (97%)	410 (96%)
Other	3 (<1%)	0 (0%)
**Age**		
Mean (SD)	43.2 (11.7)	45.4 (10.9)
Min–max	20–76	24–68
**Years of working as a nurse**		
<3 yrs.	150 (13%)	42 (10%)
3–10 yrs.	269 (23%)	90 (21%)
11–20 yrs.	350 (30%)	144 (34%)
20 yrs.<	367 (32%)	150 (35%)
**Region**		
Capital	251 (22%)	99 (23%)
Central Denmark	594 (51%)	230 (54%)
North Denmark	77 (7%)	20 (5%)
Zealand	103 (9%)	32 (8%)
Southern Denmark	110 (9%)	43 (10%)
Not registered	31 (3%)	2 (<1%)
**Department**		
Outpatient clinic	176 (15%)	80 (19%)
Anaesthetics, perioperative care or surgical areas	172 (15%)	64 (15%)
Children's ward	34 (3%)	11 (3%)
Intensive care unit	66 (6%)	39 (9%)
Surgical	150 (13%)	55 (13%)
General medicine	182 (16%)	69 (16%)
Psychiatry	41 (4%)	21 (5%)
Emergency department	87 (8%)	31 (7%)
Other	166 (14%)	52 (12%)
Not registered	92 (8%)	4 (1%)
**Current work situation**		
I have been deployed to a ward transformed into a COVID‐19 unit	266 (23%)	33 (7.7%)
I have been deployed to a ward that *does not* care for patients with COVID‐19	88 (7%)	14 (3.3%)
I have only been on my permanent ward, which has been transformed into a COVID‐19 unit	254 (22%)	125 (29.3%)
I have only been on my permanent ward that *does not* care for patients with COVID‐19	444 (38%)	243 (57.0%)
Not registered	113 (10%)	11 (3%)
**Currently caring for patients infected with COVID‐19**		
Yes	Not asked	265 (62.2%)
No	Not asked	129 (30.3%)
I do not know	Not asked	26 (6.1%)

### 3.1 COVID‐19‐related worry

More than half of the participants (57%) expressed that they had felt somewhat or highly unsafe during their work because of COVID‐19, and two‐thirds (68%) reported having been anxious for their relatives because of their job (see Table 2). The majority expressed trust in the way their management handled the situation (78%). However, an almost equal amount of the participants (71%) did not agree that the flexibility expected from nurses during the pandemic was appropriate, and 80% did not agree that nurses as a group received proper recognition for their efforts. No less than 36% had turnover intentions because of the management of COVID‐19, and another 9% was uncertain. Of those who indicated turnover intentions, 121 (81%) provided in‐depth explanations for their considerations. These could be divided into themes such as feeling unsafe because of the disease, poor well‐being, work–life balance, dissatisfaction with COVID‐19 management and considerations about job change even before the pandemic. Concerning the latter, several wrote that COVID‐19 became the trigger to truly consider resigning. Finally, 22% of those indicating turnover intentions write that they are considering altogether leaving the nursing profession (qualitative data not shown).

**TABLE 2:** Work‐related worry and attitude towards COVID‐19 in overall sample

	Number (%)	Subgroups based on dichotomized items Number (%)
I have had COVID‐19^a^	419	
Yes (I tested positive)	43 (10%)	
Yes (I believe so but not tested)	6 (1%)	
Maybe (I was not tested but I had symptoms)	7 (2%)	
No (I had no symptoms)	354 (84%)	
Do not know	9 (2%)	
I have felt unsafe in my work because of COVID‐19^a^	417	
To a high degree	60 (14%)	Highly *n* = 60 (14%)
Somewhat	179 (43%)	All others *n* = 357 (86%)
To a lesser degree	142 (34%)	
Not at all	36 (9%)	
I have been worried about my close relatives because of my work as a nurse during COVID‐19^a^	419	
To a high degree	118 (28%)	Highly *n* = 118 (28%)
Somewhat	166 (40%)	All others *n* = 301 (72%)
To a lesser degree	106 (25%)	
Not at all	29 (7%)	
I have trust in the management's handling of COVID‐19^a^	419	
To a high degree	130 (31%)	High or Some *n* = 325 (78%)
Somewhat	195 (47%)	
To a lesser degree	81 (19%)	No or Little *n* = 94 (22%)
Not at all	13 (3%)	
The flexibility expected during the COVID‐19 pandemic by nurses as a professional group is adequate^b^	418	
Strongly agree	9 (2%)	
Agree	36 (9%)	
Neither agree nor disagree	74 (18%)	
Disagree	185 (44%)	
Strongly disagree	114 (27%)	
As a professional group, nurses receive sufficient recognition for their work during the COVID‐19 pandemic^b^	418	
Strongly agree	7 (2%)	
Agree	28 (7%)	
Neither agree nor disagree	50 (12%)	
Disagree	157 (37%)	
Strongly disagree	176 (42%)	
The management of COVID‐19 in my workplace has made me consider changing jobs^b^	418	
Yes	149 (36%)	149 (36%)
No	232 (56%)	232 (56%)
I Do not know	37 (9%)	

^a^ Measured in both surveys.

^b^ Measured in the second survey.

### 3.2 Development in distress

For the overall sample, the average level of distress was slightly increased from baseline to follow‐up, although only changes in symptoms of depression and insomnia were significant and with small effect sizes (see Table 3).

**TABLE 3:** Difference in stress, and symptoms of depression, anxiety and insomnia between baseline and follow‐up

Outcome	Time point	*N*	Mean	SD	*T*	*p*	Effect size *d*
Stress PSS (0–40)	Baseline	407	13,4226	735,438	−0.844	.399	−0.042
	Follow‐up	407	13,6806	706,836			
Depression PHQ‐9 (0–27)	Baseline	414	49,879	470,355	−2.147	**.032**	−0.106
	Follow‐up	414	54,517	473,218			
Anxiety GAD‐7 (0–21)	Baseline	413	34,939	389,586	−1.381	.168	−0.068
	Follow‐up	413	37,191	386,590			
Insomnia ISI (0–28)	Baseline	414	73,285	595,124	−3.809	**<.001**	−0.187
	Follow‐up	414	82,609	619,807			

*Note*: Statistically significant values are shown in **bold**.

Abbreviations: ISI, insomnia severity index; GAD‐7, generalized anxiety disorder; PHQ‐9, patient health questionnaire; PSS, perceived stress scale.

Dividing the sample into subgroups revealed that some participants experienced a larger increase in distress than others. Number of years working as a nurse and trust in management's handling of COVID‐19 were associated with changes in distress. Nurses with more than 2 years of experience reported stable levels of distress, whereas more inexperienced nurses increased in symptoms of depression and insomnia. In addition, nurses who reported to have been highly anxious for their relatives had significantly higher increases in symptoms of depression than the remaining nurses. No other subgroup difference in development of distress were observed (see Table 4).

**TABLE 4:** Development in symptoms of stress, depression, anxiety and insomnia from baseline to follow‐up in different subgroups of nurses

	Stress Mean (SD)		Depression Mean (SD)		Anxiety Mean (SD)		Insomnia Mean (SD)	
	Baseline	FU	Baseline	FU	Baseline	FU	Baseline	FU
**Nursing experience**								
<2 yrs (*n* = 42)	16.8 (7.7)	17.6 (7.4)	6.3 (4.5)	8.6 (5.6)	5.1 (4.8)	5.9 (5.0)	8.0 (6.8)	11.1 (7.3)
>2 yrs (*n* = 369)	13.1 (7.2)	13.3 (7.4)	4.9 (4.7)	5.1 (4.5)	3.3 (3.8)	3.5 (3.7)	7.3 (5.9)	8.0 (6.0)
Time x group *F* (*p*)	0.281 (.596)		**7.768 (.006)**		1.104 (.294)		**8.103 (.005)**	
**Trust in management**								
Some/High (*n* = 325)	12.4 (7.1)	12.6 (6.9)	4.4 (4.2)	4.8 (4.5)	3.0 (3.7)	3.2 (3.7)	6.8 (5.7)	7.8 (6.0)
No/Little (*n* = 94)	16.7 (7.4)	17.2 (6.6)	7.1 (5.6)	7.6 (5.0)	5.1 (4.2)	5.5 (3.8)	9.2 (6.4)	10.0 (6.5)
Time x group *F* (*p*)	0.146 (.702)		0.004 (.949)		0.493 (.483)		0.075 (.784)	
**Feeling unsafe**								
Highly unsafe (*n* = 60)	18.2 (7.5)	18.3 (6.8)	7.8 (6.0)	8.3 (5.1)	5.8 (5.0)	6.4 (4.1)	10.5 (6.9)	11.3 (6.1)
Others (*n* = 357)	12.6 (7.0)	12.9 (6.8)	4.5 (4.3)	5.0 (4.7)	3.1 (3.6)	3.3 (3.6)	6.8 (5.6)	7.8 (6.1)
Time x group *F* (*p*)	0.037 (.849)		0.001 (.975)		0.875 (.350)		0.049 (.825)	
**Anxious relatives**								
Highly anxious (*n* = 118)	16.2 (7.6)	16.9 (6.7)	6.6 (5.1)	7.8 (5.1)	5.0 (4.6)	5.3 (4.0)	8.9 (6.6)	10.5 (6.2)
Others (*n* = 301)	12.3 (7.0)	12.4 (6.8)	4.4 (4.4)	4.6 (4.3)	2.9 (3.4)	3.1 (3.6)	6.7 (5.6)	7.4 (6.0)
Time x group *F* (*p*)	0.581 (.447)		**4.540 (.034)**		0.147 (.701)		2.855 (.092)	
**Turnover intentions**								
Yes (*n* = 147)	17.4 (7.3)	16.9 (6.6)	7.5 (5.4)	8.1 (5.0)	5.5 (4.6)	5.7 (4.1)	9.6 (6.4)	10.9 (6.4)
No (*n* = 230)	10.8 (6.2)	11.5 (6.7)	3.2 (3.1)	3.7 (3.7)	2.1 (2.6)	2.4 (3.1)	5.4 (4.8)	6.3 (5.3)
Time x group *F* (*p*)	2.744 (.098)		0.018 (.893)		0.009 (.923)		0.576 (.448)	
**COVID patients**								
Yes (*n* = 261)	13.6 (7.2)	13.8 (7.1)	4.9 (4.5)	5.6 (4.9)	3.4 (3.8)	3.7 (4.0)	7.5 (5.9)	8.4 (6.3)
No + do not know (*n* = 153)	13.2 (7.7)	13.5 (7.1)	5.1 (5.0)	5.2 (4.5)	3.6 (4.0)	3.7 (3.6)	7.1 (6.0)	8.0 (6.1)
Time x group *F* (*p*)	0.071 (.790)		1.287 (.257)		0.186 (.666)		0.017 (.896)	
**Deployed**								
Yes (*n* = 47)	14.5 (7.4)	13.6 (7.0)	5.9 (5.3)	6.0 (4.7)	3.8 (4.0)	3.8 (3.8)	7.5 (5.6)	8.4 (6.0)
No (*n* = 361)	13.3 (7.4)	13.7 (7.1)	4.9 (4.6)	5.4 (4.8)	3.5 (3.9)	3.8 (3.9)	7.3 (6.0)	8.3 (6.2)
Time x group *F* (*p*)	1.760 (.185)		0.500 (.480)		0.278 (.598)		0.017 (.896)	

### 3.3 Predictors of change in distress

We conducted a series of linear regression models to examine multivariate patterns of predictors of distress symptoms at follow‐up while controlling for baseline distress scores. These revealed that nurses with less than 2 years of experience working as a nurse reported higher levels of all kinds of distress at follow‐up. In addition, feeling unsafe and having low trust in how the management handled COVID‐19 were associated with increased anxiety, and low trust in management was also associated with higher stress levels at follow‐up. Being anxious for relatives was associated with more depressive, stress and insomnia symptoms. Neither having COVID‐19 patients at the ward nor being deployed to other departments was associated with changes in distress (see Table 5).

**TABLE 5:** Linear regression models predicting nurses' stress, depression, anxiety and insomnia symptoms at follow‐up

	Change in stress Model *F* = 43.501*** Adj. *R* ^2^ = 0.431 (Standardized coefficients Beta)	Change in depression Model *F* = 34.131*** Adj. *R* ^2^ = 0.368 (Standardized coefficients Beta)	Change in anxiety Model *F* = 44.326** Adj. *R* ^2^ = 0.432 (Standardized coefficients Beta)	Change in insomnia Model *F* = 48.831** Adj. *R* ^2^ = 0.456 (Standardized coefficients Beta)
Baseline distress^a^	.559***	.491***	.577***	.626***
Anxious for relatives	.098*	.103*	.016	.092*
Feeling unsafe	.067	.067	.104*	.032
Trust in management	−.097*	−.084	−.093*	−.015
0‐2 yrs nurse versus >2 yrs nurse	−.091*	−.159***	−.102**	−.119**
Deployed at other ward	−.034	−.002	−.009	−.006
COVID patients at ward	−.022	.010	−.009	−.021

^a^ Baseline distress score is entered in the respective model.

* *p* < .05.

** *p* < .01.

*** *p* < .001.

### 3.4 Turnover intentions

Finally, we tested whether the same parameters predicted nurses' turnover intentions, and we found that being anxious for relatives, feeling unsafe and having little trust in management, but not years of experience working as a nurse, being deployed at other wards or having COVID‐19 patients predicted intention to change job (see Table 6).

**TABLE 6:** Predictors of turnover intentions in binary logistic model

	Model Chi^2^ = 71.513 *p* < .001	95% Confidence interval	
	Odds ratio	Lower	Upper
Anxious for relatives			
Low	1		
High	2.529	1.440	4.443
Feeling unsafe			
Low	1		
High	2.726	1.374	5.411
Trust in management			
Low	1		
High	3.837	2.190	6.722
Work experience			
0–2 yrs. nurse	1		
>2 yrs. nurse	0.618	0.282	1.352
Deployed at other ward			
No	1		
Yes	1.222	0.581	2.569
COVID patients at ward			
No	1		
Yes	1.569	0.961	2.562

## 4 DISCUSSION

To our knowledge, this is the first study to report on longitudinal mental health in a Danish sample of hospital‐based nurses 8 months after the COVID‐19 outbreak in Denmark. The study finds that the mental health of nurses in Danish hospitals during the beginning of the pandemic was strained, as the nurses reported distress in the form of symptoms of depression, anxiety, stress and sleep problems. The results are in line with several international studies investigating the impact of the pandemic on nurses' mental health, which demonstrates that nurses around the world have been mentally burdened by the pandemic (Pappa et al., 2020; Sanghera et al., 2020; Vizheh et al., 2020). A significant proportion of the nurses in our study reported having felt unsafe at work and even more had worried about unintentionally infecting their relatives. These findings have also been found and described qualitatively in studies exploring the experiences of nurses during the COVID‐19 pandemic (Galehdar et al., 2020; Liu et al., 2020). For the participants to be distressed by worries both at work (feeling unsafe) and at home (anxious about relatives) poses an additional burden in relation to their mental health, because there is no place to recover.

Although several studies have examined the psychological impact of working as a nurse during the COVID‐19 pandemic, only a small number have evaluated the impact over time, and of those, most have short follow‐up periods (Abdalla et al., 2021; Sampaio et al., 2021). One of the few studies with a long follow‐up period found an overall decrease in depression, anxiety and post‐traumatic stress symptoms among Italian health professionals 14 months into the pandemic. Although they observed an overall worsening in insomnia symptoms, the increase was not clinically relevant (Rossi et al., 2021). Although COVID‐19 appeared to have a substantial and immediate negative impact on the mental health of nurses, the results of our study and the study by Rossi et al. suggest that for the majority of health professionals, including nurses, a psychological adaptation is taking place over time. A few studies even observed positive variation in psychological distress during the initial months of the pandemic. A Portuguese study found decrease in distress concerning sleep quality and symptoms of depression, anxiety and stress, whereas a study on sleep quality among health professionals found that the initial high rates of insomnia symptoms improved as more time passed from the peak of local COVID‐19 cases 10 weeks after baseline (Abdalla et al., 2021; Sampaio et al., 2021). However, in our study, although a psychological adaptation may have taken place, we found that the baseline level of distress was elevated, indicating that the participating nurses were already burdened early in the pandemic, and this did not improve. In the wake of past pandemics, it has been pointed out that the role of nursing management is crucial in supporting initiatives to address the mental health of nurses who have been under enormous strain (Lau & Chan, 2005). In our study, inexperienced nurses and those with low or no trust in management increased in distress during the first 8 months of the pandemic. These results highlight the importance of long‐term follow‐up to respond to and ease the potential continuing mental health impact of the pandemic. A recent two‐year impact assessment among 12,000 American nurses finds that younger nurses and nurses with less experience struggle more with poor mental health than older and more experienced nurses. The study also found that almost two‐thirds of nurses under the age of 25 and more than half of nurses aged 25–34 do not believe their organization cares about their well‐being and generally feel unsupported (COVID‐19 Impact Assessment Survey ‐ The Second Year, 2022). Special attention should be paid to newly qualified nurses and nurses who indicated low trust in how management handled COVID‐19.

Concerns related to turnover intentions among nurses continue to challenge health‐care managers, and they are influenced by many interrelated factors (Hayes et al., 2012). Our study provides empirical support for the association between COVID‐19‐related worries and turnover intentions. More than a third of the nurses had turnover intentions explained by the management's handling of COVID‐19, and because nurses' turnover intention is a solid predictor of actual nurse turnover, that may give rise to concern (Yang et al., 2017). We found that feeling unsafe at work, worried about relatives because of one's work as a nurse and low trust in management's handling of COVID‐19 significantly predicted the participants' considerations about job turnover. In a study by Labrague and de Los Santos, they found that after adjusting for nurse and hospital characteristics, an increased level of fear of COVID‐19 was associated with increased psychological distress and turnover intentions (Labrague & de Los Santos, 2021). A recent review found that following the outbreak of COVID‐19, the most reported factors predicting turnover intention among nurses included fear of the disease, stress and anxiety (Falatah, 2021). The review also examined factors predicting turnover intention prior to the COVID‐19 pandemic, and the findings included satisfaction, commitment and leadership style (Falatah, 2021). We found that low trust in management's handling of COVID‐19 predicted turnover intentions. Participating nurses' elaborations in the open text box, however, indicated that several had already considered job change before the pandemic because of dissatisfaction with management, and the extra pressure in the form of more shifts and involuntary deployment to other departments had merely confirmed them to really consider a turnover. Previous research on workplace‐specific job attributes and voluntary employee turnover has shown that if the employer does not take the necessary measures to improve work conditions, employees will retain their intentions to change jobs (Cottini et al., 2011). Moreover, in a study of leadership styles among nurse managers, analysis revealed that participative and transformational leadership styles decreased turnover intention, whereas autocratic and laissez‐faire leadership styles increased turnover intention (Magbity et al., 2020). Hence, the importance of poor work conditions, management and their relation to turnover intentions cannot be underestimated.

### 4.1 Study limitations

Despite its longitudinal design, this survey has some limitations. Online surveys generally permit convenient and cost‐effective research. However, the distribution through social media may have resulted in sampling bias. Although the anonymity of online surveys may be preferable for the participants, it creates methodological weaknesses as it makes non‐response analyses impossible. We used targeted advertising on social media to increase the survey's visibility to the target population. However, the survey may have been primarily completed by nurses who had a strong opinion regarding the topic. Moreover, two‐thirds of the participants from the baseline survey did not respond to the follow‐up. Hence, the findings cannot be generalized. Another limitation was that we did not have data on the participants prior to COVID‐19. Therefore, the results should be interpreted with caution as we do not know if the participants had symptoms of distress or turnover intentions before the pandemic. However, a study conducted among intensive care unit professionals in the Netherlands found an increase in burnout symptoms in the post‐peak COVID‐19 period, which on a timeline corresponds to the baseline in the present study, compared to before the outbreak (De Kock et al., 2021). Also, with turnover intentions, we asked the participants to describe why they were considering a job change and received 121 individual and mainly extended answers shedding light on why some of the 35% considered job changes.

## 5 CONCLUSION

The present follow‐up study found that exposure to work with COVID‐19 patients or being deployed to another ward was not associated with increased distress in nurses. Feeling unsafe, being anxious for relatives, having low trust in management and limited experience as a nurse was associated with increased distress. This suggests that the subjective experiences of feeling unsafe in work under the COVID‐19 pandemic have a larger impact on nurses' distress than the organizational conditions at hospitals. Moreover, the study provides empirical support for the association between COVID‐19‐related worries and turnover intentions.

## 6 IMPLICATIONS FOR NURSING MANAGEMENT

Poor mental health and job burnout among nurses have been a crucial issue during the COVID‐19 pandemic. Hospital management needs to be aware of and act on the mental well‐being of health‐care professionals in order to support those in need to overcome distress. Knowledge of risk factors for psychological distress and burnout is needed and may provide nurses and health‐care systems with the ability to prioritize and respond better against pandemics or other health crises in the future.

Although current explanations for turnover intentions can be found in the light of COVID‐19, it does not change the fact that the health‐care system faces important tasks. The increasing pressure on the health‐care system, combined with the fact that more nurses are considering leaving the profession, can lead to critical challenges. Therefore, identification of predictors of turnover intention would enable managers to direct attention to work‐related changes in health‐care organizations in order to retain nurses in the organization and in the profession.

## CONFLICT OF INTEREST

The authors declared no potential conflicts of interest with respect to the research, authorship and/or publication of this article.

## ETHICS STATEMENT

According to Danish law, this kind of self‐reported questionnaire study does not require notification to the Committee on Biomedical Research Ethics. All procedures performed in studies involving human participants were in accordance with the ethical standards of the institutional and national research committee and with the 1964 Helsinki declaration and its later amendments or comparable ethical standards. Informed consent were obtained and all general requirements for health science research were followed.

## Data Availability

The data that support the findings of this study are available from the corresponding author upon reasonable request.
